# The Action of the Philadelphia Dental College

**Published:** 1868-05

**Authors:** 


*The Action of the Philadelphia Dental College.*—The Association of the Colleges of Dentistry met in the city of New York on the second day of April last and the following Colleges were represented. Baltimore, Ohio, New York, and Missouri. The Penn. College of Dental Surgery having withdrawn at the last meeting held in Philadelphia, no delegates were expected to represent this College. But from the fact that the Philadelphia Dental College had been one of the first to take an active part in the formation of this Association, and one of the most zealous in its former meetings, expressing as much, if not more, indignation at the action of the seceeding College, as any of the other Colleges, it will no doubt surprise many of our readers to learn that on the second day of the meeting of the Association the following note was received from the Dean: "Resolved that the Faculty of the Philadelphia

Dental College respectfully withdraws its connection with the Association of the Colleges of Dentistry." "Resolved that the Dean be instructed to transmit this resolution to the above Association."

The members of the Association, although rumors of dissatisfaction on the part of this College had reached them before the receipt of the communication, had expected a totally different course from an institution which had shown such a strong desire to improve the system of dental education. It would certainly have been more to the credit of the Faculty of this College had one of its delegates been present at the meeting of the Association, and in a straightforward, open manner stated their objections to the rules for which their votes had been cast, and endeavored to have such as they regarded as being injurious to their institution modified. But adopting no such course they withdraw from the Association by a written notification which gives no reasons for such withdrawal.

We owe to those members of the dental profession who wish to elevate its standard, the following explanation of the cause of this withdrawal on the part the Phila. College. At the last session of this College 1867-68, the number of matriculants was 44, and the number of graduates 20, against 70 matriculants and 30 graduates for the previous session of 1866-67. Attributing this falling off in numbers to their observance of the rules adopted by the Association, all of which, if not proposed by their delegates, were voted for by them, and also from the fact that their rival the Penn. School had a greater number of both matriculants and graduates, the Phila. Dental College concluded that it was preferable to have a larger than a better class; hence their action.

The Baltimore, Ohio, New York and Missouri Colleges all suffered as regards the number, but not, we feel confident, as regards the quality of the students attending their lectures last session. The class of the Baltimore College during the last session would have numbered 85 instead of 69, had students been admitted on the old rules; but yet this College, and also the others forming the Association, prefer having a smaller number of good students to a larger number of indifferent ones.

We could not pass this matter over without notice, as it is one which challenges the censure or approval of the Profession.

